# Intraoperative blood transfusion increases the incidence of acute kidney injury in elective off pump coronary artery surgery

**DOI:** 10.1186/1749-8090-10-S1-A168

**Published:** 2015-12-16

**Authors:** Prabhat Tewari, Shantanu Pandye, Surendra Kumar Agarwal

**Affiliations:** 1 Department of Anaesthesiology, SGPGIMS, Raibarely Road, Lucknow, Uttar Pradesh, 226014, India; 2 Department of Cardiothoracic and Vascular Surgery, SGPGIMS, Raibarely Road, Lucknow, Uttar Pradesh, 226014, India

## Background/Introduction

Acute kidney injury (AKI) occurs in up to 30% of patients undergoing coronary artery bypass grafting. Development of kidney injury is associated with high overall mortality and a more complicated hospital course. Cardiopulmonary bypass is considered as one of the major risk factors for developing AKI. Off-pump coronary artery bypass (OPCAB) grafting eliminates the need for cardiopulmonary bypass and, as such, is assumed to reduce AKI. However, previous studies provided conflicting evidence to support this hypothesis.

## Aims/Objectives

We tried to find out whether intraoperative blood transfusions during OPCAB surgery affects renal functions in the postoperative period and also effects of existing preoperative anaemia and renal dysfunction.

## Method

850 first time patients undergoing OPCAB surgery were prospectively included. Baseline and intraoperative haematocrit (Hct) were recorded. The transfusion trigger was Hct of 24 as institutional practice. Those in whom the Hct level touched 24 or less were randomized to either Gr. A (receiving blood transfusion of two units of packed cells) or Gr. B (no blood transfusion during surgery). Postoperative haemodynamic assessment, creatinine levels, urea levels, haemoglobin levels, blood gas analysis for acidosis and serum potassium levels, urine output, ICU stay, days to discharge, IABP use were compared. Data were presented as mean with SD and compared (SPSS).

## Results

In 55 patients the Hct touched the nadir and they were randomized to Gr A (28 patients) and Gr. B. (22 patients)that were investigated completely. In postoperative period, in Gr. A 8 patients (29%) developed AKI whereas in Gr. B 1 patient had AKI. Out of nine, 6 patients underwent haemodialysis and there was 1 mortality in Gr. A. Out of these 8 patients 4 have pre op raised creatinine and in group B the single patient has raised creatinine.

## Discussion/Conclusion

Intraoperative Blood transfusion seems to be an independent risk factor to cause AKI in postoperative period in first time off pump surgery and the incidence is more in the setting of pre-existing anaemia and renal dysfunction. To conclude it seems to be better to avoid blood transfusions during intraoperative period as persisting haemodynamic upheavals during manipulation of heart make kidneys more prone to injury.

